# Short-term Outcomes of Corticosteroid Monotherapy in Multisystem Inflammatory Syndrome in Children

**DOI:** 10.1001/jamapediatrics.2022.0292

**Published:** 2022-03-28

**Authors:** D. Sofia Villacis-Nunez, Kaitlin Jones, Aysha Jabbar, Lucie Fan, Whitney Moore, Andrew S. Peter, Michaela Henderson, Yijin Xiang, Michael S. Kelleman, Whitney Sherry, Shanmuganathan Chandrakasan, Matthew E. Oster, Preeti Jaggi, Sampath Prahalad

**Affiliations:** 1 Division of Pediatric Rheumatology, Department of Pediatrics, Emory University School of Medicine, Atlanta, Georgia; 2 Children’s Healthcare of Atlanta, Atlanta, Georgia; 3 Emory University School of Medicine, Atlanta, Georgia; 4 Sibley Heart Center, Atlanta, Georgia; 5 Pediatrics Biostatistics Core, Atlanta, Georgia; 6 Division of Hospital Medicine, Department of Pediatrics, Emory University School of Medicine, Atlanta, Georgia; 7 Division of Hematology, Oncology and BMT, Department of Pediatrics, Emory University School of Medicine, Atlanta, Georgia; 8 Division of Infectious Diseases, Department of Pediatrics, Emory University School of Medicine, Atlanta, Georgia; 9 Department of Human Genetics, Emory University School of Medicine, Atlanta, Georgia

## Abstract

**Importance:**

Optimal agents and duration of primary treatment for multisystem inflammatory syndrome in children (MIS-C) remain unclear.

**Objective:**

To compare short-term patient outcomes based on initial treatment with corticosteroids, intravenous immunoglobulin (IVIG), or both.

**Design, Setting, and Participants:**

This retrospective cohort study included patients in a tertiary-care pediatric hospital system who had MIS-C per the Centers for Disease Control and Prevention case definition during the period March 2020 to February 2021.

**Exposures:**

Immunomodulatory therapy within the first 24 hours (patients in the intensive care unit [ICU]) or 48 hours (non-ICU patients): corticosteroids alone, IVIG alone, and IVIG plus corticosteroids.

**Main Outcomes and Measures:**

Primary outcome was failure of initial therapy, defined as therapy escalation due to fever or worsening or lack of improvement of laboratory, cardiac, or noncardiac clinical factors after 24 hours (ICU patients) or 48 hours (non-ICU patients) from time of therapy initiation, per clinician assessment. Secondary outcomes included presence of complications, cardiovascular outcomes, fever duration, length of hospital and ICU stays, corticosteroid use duration, and need for readmission.

**Results:**

Among 228 eligible patients, 215 patients were included in the univariate analysis; median age was 8 years, and 135 (62.8%) were boys. There were 69 patients in the corticosteroids group, 31 patients in the IVIG group, and 115 patients in the IVIG plus corticosteroids group. Patients in the corticosteroids group had milder disease at presentation. After propensity score weighting including 179 patients (68 in the corticosteroids group and 111 in the IVIG plus corticosteroids group), rates of initial treatment failure were similar between groups. Among patients whose initial treatment failed, treatment failure in the IVIG plus corticosteroids group was more likely to be based on laboratory parameters (odds ratio [OR], 1.96; 95% CI, 1.07-3.60) and less likely to be based on cardiovascular markers (OR, 0.39; 95% CI, 0.2-0.76), per clinician assessment. Patients in the IVIG plus corticosteroids group had a longer median inpatient stay (6 vs 5 days; *P* = .001) and longer median corticosteroid course duration (10 vs 5 days; *P* = .04) compared with the corticosteroids group. Forty-nine patients (71% of 69 in the corticosteroids group) recovered after receiving corticosteroid monotherapy for 10 days or less.

**Conclusions and Relevance:**

Corticosteroid monotherapy is a reasonable management option for a subset of patients with MIS-C, particularly those with mild disease.

## Key Points

**Question:**

Is corticosteroid monotherapy a viable treatment alternative for multisystem inflammatory syndrome in children (MIS-C)?

**Findings:**

In this cohort study, patients receiving corticosteroids as initial management had similar rates of treatment failure compared with those receiving intravenous immunoglobulin plus corticosteroids, after adjusting for baseline presentation and disease severity; in the latter group, therapy failure due to laboratory parameters was more likely while failure due to cardiac parameters was less likely. Inpatient stay and corticosteroid course duration were shorter in patients initially treated with corticosteroid monotherapy.

**Meaning:**

In this study, corticosteroid monotherapy successfully treated a subset of patients with mild MIS-C.

## Teaser

This cohort study compares initial treatments for multisystem inflammatory syndrome in children to assess short-term patient outcomes associated with corticosteroids, intravenous immunoglobulin, and both.

## Introduction

Multisystem inflammatory syndrome in children (MIS-C) is an uncommon complication of SARS-CoV-2 infection, characterized by fever, multiorgan involvement, laboratory evidence of inflammation, and documented SARS-CoV-2 exposure. Approximately 80% of cases present cardiovascular symptoms and 74% develop mucocutaneous features, resembling Kawasaki disease (KD), with potential development of coronary artery abnormalities.^1,2^

The pathophysiology of MIS-C is not completely understood. Autoreactivity and postviral dysregulated immune activation have been suggested as major drivers of tissue damage.^3^ Thus, besides inotropic and respiratory support, addressing the inflammatory response through immunomodulation is essential.^2^ Because of their similarities, the management of MIS-C mirrors the approach for KD.^2,4^ However, compared with KD, patients with MIS-C often exhibit more robust inflammatory responses and higher rates of intravenous immunoglobulin (IVIG) resistance, which may call for early use of broader-spectrum immunosuppressants such as corticosteroids.^4,5,6,7^ The efficacy of these interventions is still under analysis, and in the absence of randomized clinical trial results, observational studies remain fundamental. This study compares short-term patient outcomes based on initial immunomodulation and describes our experience with corticosteroid monotherapy, which could help guide treatment for future cases during this ongoing pandemic.

## Methods

For this retrospective cohort study, we identified children diagnosed with MIS-C between March 1, 2020, and February 28, 2021, at Children’s Healthcare of Atlanta, a health care system comprising 3 freestanding children’s hospitals in metropolitan Atlanta, Georgia. Some patients in March and early April 2020 were diagnosed retrospectively after the case definition was established. Starting in May 2020, we began maintaining a prospectively collected database of patients diagnosed with MIS-C. Cases were identified through daily surveillance using the Centers for Disease Control and Prevention case definition^8^ and adjudicated weekly with a multidisciplinary group discussion, after consideration of alternative etiologies. Exclusion criteria included use of anticytokine therapy in the first 24 hours, no treatment administration, pretreatment at another facility, or therapy escalation for an unclear reason. Demographic information (including family-reported race and ethnicity) and clinical variables not included in the original database were retrospectively collected. Race and ethnicity data were collected because they are known to influence COVID-19 severity and could similarly affect outcomes in MIS-C. Race categories included African American, White, and other, which included any response that did not fit into the other 2 categories. Ethnicity categories included Hispanic or Latino and non-Hispanic or Latino. The study was approved by the Children’s Healthcare of Atlanta institutional review board via waiver of consent.

The time of administration of the first dose of IVIG or corticosteroids was considered the time of therapy initiation. Three groups were defined based on the initial therapy received: corticosteroids alone, IVIG alone, or IVIG plus corticosteroids. Initial therapy was considered IVIG plus corticosteroids when the administration of each medication occurred within 24 hours of one another for patients in the intensive care unit (ICU) or within 48 hours for non-ICU patients.

Primary and secondary outcomes were used to evaluate treatment response. The primary outcome was failure of initial therapy, defined as a patient receiving additional treatment as documented in the medical record by the treating clinician for fever, worsening or lack of improvement of laboratory markers, worsening or lack of improvement of cardiac findings, and/or worsening or lack of improvement of noncardiac clinical findings, after 24 hours (ICU patients) or 48 hours (non-ICU patients) from the time of therapy initiation. This definition was intended to reflect current clinical practice at our centers, where clinicians determine therapy failure and addition of adjuvant immunosuppressants based on the aforementioned factors and base the timing of this determination on whether the patient’s condition is considered critical (ICU status). Secondary outcomes included presence of complications, cardiovascular outcomes, fever duration, length of hospital and ICU stay, corticosteroid therapy duration, and readmission within 6 months of diagnosis. eTable 1 in the Supplement lists complete definitions used to delineate baseline characteristics (at the start of immunomodulatory treatment), medication-related variables, adverse events potentially related to medications, and primary and secondary outcomes.

Categorical variables are expressed as frequency (percentage) and continuous variables as median (IQR). Unadjusted 2-group comparisons were conducted using Fisher or χ^2^ test for categorical variables and Wilcoxon rank sum test for continuous variables.

The IVIG group was excluded from adjusted analysis because of the small sample size. Imbalances in baseline features between the corticosteroids and IVIG plus corticosteroids groups were identified using standard mean differences (SMDs). Demographics, baseline clinical variables, and factors indicative of severe disease, particularly those with SMDs greater than 0.20, were identified as potentially confounding of exposure-outcome associations. Propensity scores were calculated using logistic regression, and inverse probability of treatment weighting (IPTW) methods were applied to balance these potentially confounding characteristics between the groups and decrease the effect of treatment-selection bias.^9^ See eTable 2 in the Supplement for a list of variables and scores included in these calculations. Extreme weights were truncated at the first and 99th percentiles. Missing rates were less than 5% for all variables; patients with missing data were excluded from the final models. We calculated IPTW-adjusted odds ratios (OR) for categorical variables and IPTW-adjusted least-squares means (LS-means) for continuous variables, with corresponding 95% CI. *P* values of .05 and less were considered statistically significant. All analyses were performed using SAS version 9.4 (SAS Institute) and R version 4.0.2.

## Results

### Baseline Characteristics of the Study Cohort

**Figure 1.**
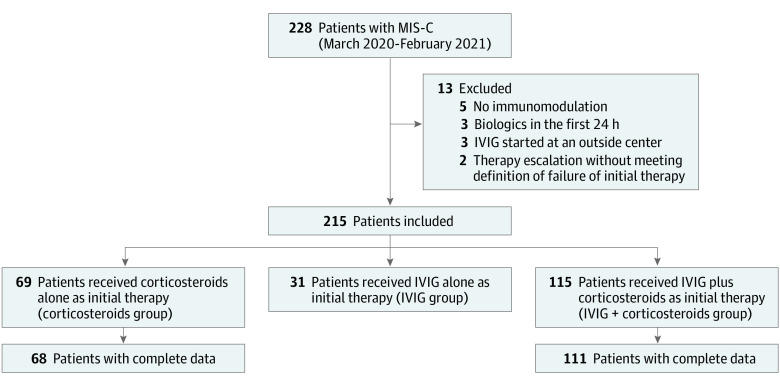
Study Cohort and Group Designation Based on Initial Therapy IVIG indicates intravenous immunoglobulin; MIS-C, multisystem inflammatory syndrome in children.

There were 228 patients with MIS-C identified within the study period, of whom 215 were included after 13 met exclusion criteria (Figure 1). Median (IQR) age was 8 years (5-12). Most patients were male (62.8%) and African American (54.9%). Eighty-nine patients (41.4%) had comorbid conditions; obesity (n = 64) was most common. At the start of therapy, 120 patients (55.8%) were in the ICU. Hematologic, gastrointestinal, and cardiovascular were the most common systems involved, in 212 (98.6%), 206 (95.8%), and 204 (94.9%) patients, respectively. SARS-CoV-2 immunoglobulin G antibody was positive in 207 patients (98.6%). Sixty-nine patients (32.1%) received corticosteroids alone, 31 patients (14.4%) received IVIG alone, and 115 patients (53.5%) received IVIG plus corticosteroids as initial therapy for MIS-C.

eTable 3 in the Supplement details demographics, presenting clinical features and initial laboratory values for all groups. Patients in the IVIG group were younger than patients in the corticosteroids group. The IVIG group patients were less frequently White and more frequently African American. Patients in the corticosteroids group were less frequently in the ICU at the time of therapy initiation. Patients in the IVIG plus corticosteroids group had more extensive organ involvement. Specifically, IVIG plus corticosteroids group patients had higher frequency of respiratory, ocular, and cardiovascular involvement compared with the corticosteroids group and more neurologic manifestations compared with the IVIG group. Rates of kidney involvement were lowest in the corticosteroids group. Seven patients required mechanical ventilation (6 patients in IVIG plus corticosteroids group [5%], 1 patient in IVIG group [3%], and none in the corticosteroids group). The median time from fever and symptom onset to therapy initiation was longest in the IVIG group.

Cardiovascular features were less prominent in the corticosteroids group, which had higher median initial left ventricular ejection fraction (LVEF), lower frequency of pericardial effusion, and less vasoactive use compared with the IVIG and IVIG plus corticosteroids groups. Similarly, the corticosteroids group had lower rates of admission LVEF less than 55% compared with the IVIG plus corticosteroids group. Patients in the corticosteroids group had higher median levels of hemoglobin, albumin, and ferritin; lower median levels of D-dimer level and brain natriuretic peptide; and more frequent SARS-CoV-2 polymerase chain reaction positivity compared with the IVIG group. The IVIG plus corticosteroids group had lower median platelet count and higher median levels of D-dimer, ferritin, and brain natriuretic peptide compared with the corticosteroids group.

### Adverse Events Potentially Related to Medications and Therapies

**Figure 2.**
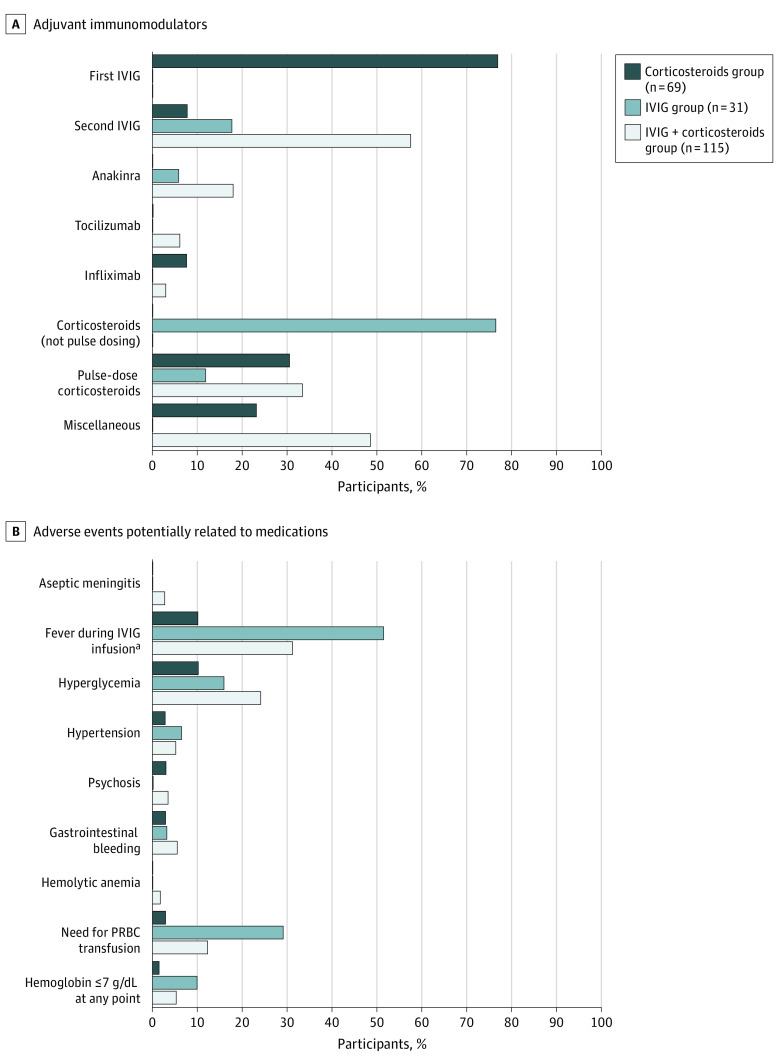
Adjuvant Therapies and Adverse Events Potentially Related to Medications A, Adjuvant immunomodulators used for patients whose initial therapy failed in all groups. Miscellaneous includes hydroxychloroquine (n = 1), increased corticosteroid dose (not pulse dosing) (n = 12), reinitiation of corticosteroid therapy for symptom recurrence after discontinuation (n = 4), and a switch to methylprednisolone from dexamethasone (n = 2). B, Rates of adverse events in all groups. IVIG indicates intravenous immunoglobulin; PRBC, packed red blood cells. ^a^Among patients receiving IVIG as adjunctive therapy (n = 10).

Figure 2 and eTable 4 in the Supplement summarize adverse events potentially related to medications and additional treatment-related details, including adjuvant immunosuppressant medications used after failure of initial treatment, antiplatelet and anticoagulant agents, and initial and maximum corticosteroid doses for all groups. Fever during IVIG infusion and hyperglycemia were the most common adverse events. Hyperglycemia was more common in the IVIG plus corticosteroids group compared with the corticosteroids group; among 40 patients with hyperglycemia, 4 patients (10%) required inpatient insulin, which was continued postdischarge in 1 patient.

Hemolytic anemia rates were low. Median hemoglobin variation value was lowest in the corticosteroids group; among IVIG recipients (both as initial or adjuvant therapy), median hemoglobin decrease after first IVIG was lowest in the corticosteroids group. Anemia requiring transfusion of packed red blood cells was most frequent in the IVIG group. Median nadir hemoglobin value was highest in the corticosteroids group.

### Outcomes

eTable 5 in the Supplement summarizes primary and secondary outcomes for all groups before propensity score weighting. Failure of initial therapy was most frequent in the IVIG group (17/31, 54.8%), followed by the IVIG plus corticosteroids group (33/115, 28.7%). Among 69 patients who received corticosteroids as initial treatment, 13 (18.8%) required additional therapy and 2 (2.9%) needed ICU transfer within 24 hours of therapy initiation. Three patients (4.3%) were readmitted (1 patient [1.4%] after initial therapy failed). Therefore, 52 patients (75.4%) recovered uneventfully. Forty-nine patients (71%) received corticosteroid monotherapy for 10 days or less.

One patient in the IVIG plus corticosteroids group required extracorporeal membrane oxygenation. Three individuals developed giant coronary aneurysms: 2 patients in IVIG plus corticosteroids group (maximum *z* score: +13.8 and +17, respectively), and 1 patient in the CS group (maximum *z* score: +31.4). The initial presentation of the latter patient was most concerning for acute SARS-CoV-2, with overlapping KD and MIS-C features and late cardiac manifestations, leading to diagnostic challenges and delay in optimal therapy (see Villacis-Nunez et al^10^ for details). Additional relevant echocardiographic findings at any point during the illness are reported in eTable 6 in the Supplement; because these variables partially contained admission data used for propensity score calculation, they are not reported in the adjusted analysis.

All patients survived. Readmissions (within 6 months of diagnosis) were due to MIS-C symptoms (1 in the corticosteroids group, none in the IVIG group, and 3 in the IVIG plus corticosteroids group), SARS-CoV-2–induced encephalopathy (1 in the IVIG plus corticosteroids group, none in the other groups), and other causes (n = 5); eTable 7 in the Supplement contains additional details pertaining these patients.

**Figure 3.**
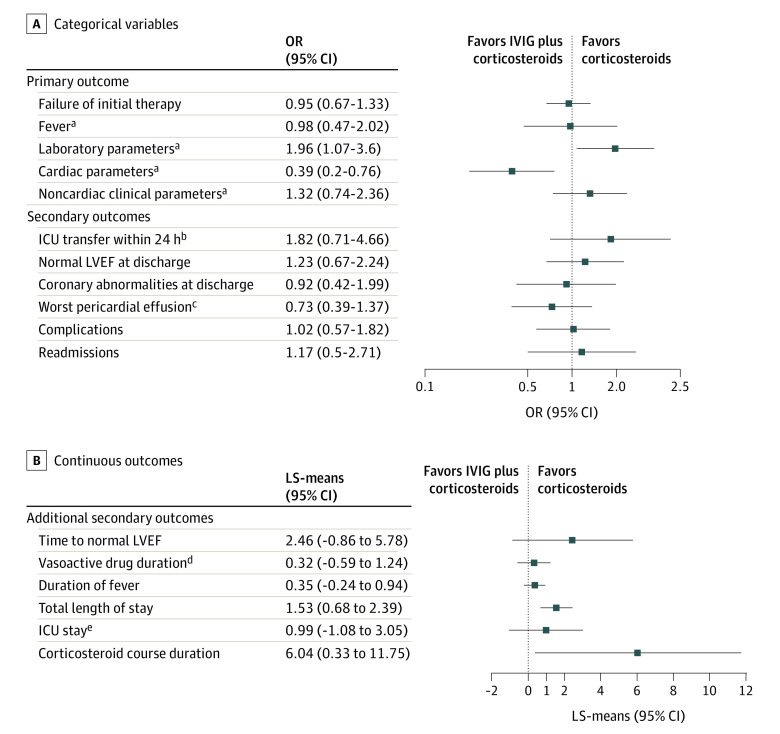
Outcome Analysis After Controlling for Potential Confounders via Inverse Probability of Treatment Weighting A, Odds ratios (ORs) and 95% CI for categorical outcomes, using the corticosteroids group as the reference group. B, Least-square means (LS-means) of continuous outcomes with their corresponding 95% CIs. ICU indicates intensive care unit; LVEF, left ventricular ejection fraction. ^a^Reasons for therapy failure among patients whose initial therapy failed. ^b^Among patients not in the ICU at the start of therapy. ^c^Mild to moderate vs trivial; among patients with a pericardial effusion. ^d^Among patients requiring vasoactive medications. ^e^Among patients who required ICU care.

Adjusted analysis using propensity score weighting included 179 patients with complete data, 68 in the corticosteroids group and 111 in the IVIG plus corticosteroids group; the IVIG group was excluded because of its small sample size. Several baseline characteristics differed between the groups, but successful application of IPTW methods balanced potential confounders (Table 1). Primary and secondary outcomes for both groups, with their corresponding pre-IPTW and post-IPTW ORs and LS-means are detailed in Table 2. Figure 3 depicts post-IPTW ORs and LS-means for all outcomes.

**Table 1.** Propensity Score Weighting Summary^a^

Baseline characteristics	Before propensity score weighting			After propensity score weighting		
	IVIG + corticosteroids (n = 111)	Corticosteroids (n = 68)	SMD	IVIG + corticosteroids (n = 116.4)	Corticosteroids (n = 61.2)	SMD
Age, y			0.202^b^			0.077
<5	25 (22.5)	10 (14.7)		22.5 (19.4)	10.0 (16.4)	
≥5	86 (77.5)	58 (85.3)		93.9 (80.6)	51.1 (83.8)	
Race			0.267^b^			0.114
African American	62 (55.9)	29 (42.6)		58.5 (50.3)	29.4 (48.0)	
White	38 (34.2)	30 (44.1)		44.7 (38.4)	26.4 (43.2)	
Other or refused	11 (9.9)	9 (13.2)		13.1 (11.3)	5.4 (8.8)	
Ethnicity			0.092			0.122
Hispanic or Latino	25 (22.5)	18 (26.5)		30.3 (26.1)	12.8 (20.9)	
Not Hispanic or Latino	86 (77.5)	50 (73.5)		86.1 (73.9)	48.4 (79.1)	
Comorbidities			0.125			0.154
No	64 (57.7)	35 (51.5)		58.3 (50.1)	25.9 (42.4)	
Yes	47 (42.3)	33 (48.5)		58.1 (49.9)	35.2 (57.6)	
Time from fever onset to therapy initiation, d			0.048			0.051
<5	61 (55.0)	39 (57.4)		63.4 (54.4)	31.8 (51.9)	
≥5	50 (45.0)	29 (42.6)		53.0 (45.6)	29.4 (48.1)	
System involvement			0.66^b^			0.024
≤3 Organ systems	42 (37.8)	47 (69.1)		62.4 (53.6)	33.5 (54.8)	
>3 Organ systems	69 (62.2)	21 (30.9)		54.0 (46.4)	27.6 (45.2)	
Platelet count			0.493^b^			0.012
<150 × 10^3^/mL	57 (51.4)	19 (27.9)		44.5 (38.2)	23.0 (37.7)	
≥150 × 10^3^/mL	54 (48.6)	49 (72.1)		71.9 (61.8)	38.1 (62.3)	
Left ventricular ejection fraction <55%			0.424^b^			0.014
No	71 (64.0)	56 (82.4)		85.6 (73.5)	44.6 (72.9)	
Yes	40 (36.0)	12 (17.6)		30.8 (26.5)	16.6 (27.1)	
Coronary abnormalities			0.046			0.121
No	105 (94.6)	65 (95.6)		109.0 (93.6)	58.9 (96.3)	
Yes	6 (5.4)	3 (4.4)		7.4 (6.4)	2.3 (3.7)	
Vasoactive use			0.864^b^			0.037
No	51 (45.9)	57 (83.8)		72.7 (62.5)	39.3 (64.3)	
Yes	60 (54.1)	11 (16.2)		43.7 (37.5)	21.9 (35.7)	
Intensive care status			0.674^b^			0.049
No	38 (34.2)	45 (66.2)		49.9 (42.9)	27.7 (45.4)	
Yes	73 (65.8)	23 (33.8)		66.4 (57.1)	33.4 (54.6)	
Severe inflammation			0.326^b^			0.049
No	19 (17.1)	21 (30.9)		24.8 (21.3)	14.3 (23.4)	
Yes	92 (82.9)	47 (69.1)		91.5 (78.7)	46.9 (76.6)	

Abbreviations: IVIG, intravenous immunoglobulin; SMD, standard mean difference.

^a^ All variables are expressed as frequency (percentage).

^b^ Value indicates significant imbalance between groups.

**Table 2.** Exposure-Outcome Associations for Corticosteroids and IVIG Plus Corticosteroids Groups Before and After Propensity Score Weighting^a^

Outcomes	Before propensity score weighting				After propensity score weighting			
	IVIG + corticosteroids (n = 111)	Corticosteroids (n = 68)	OR/LS-means (95% CI)^b^	*P* value	IVIG + corticosteroids (n = 116.4)	Corticosteroids (n = 61.2)	OR/LS-means (95% CI)^b^	*P* value
**Primary outcome**								
Failure of initial therapy	31 (27.9)	13 (19.1)	1.28 (0.89 to 1.85)	.19	32 (27.5)	18.17 (29.7)	0.95 (0.67 to 1.33)	.95
Reasons for failure of initial therapy^c^								
Fever	22 (71.0)	11 (84.6)	0.67 (0.29 to 1.56)	.35	25.58 (80.0)	14.7 (80.7)	0.98 (0.47 to 2.02)	.95
Laboratory parameters	20 (64.5)	7 (53.8)	1.25 (0.65 to 2.41)	.51	21.3 (66.6)	6.2 (34.2)	1.96 (1.07 to 3.6)	.03^d^
Cardiac parameters	15 (48.4)	7 (53.8)	0.9 (0.47 to 1.72)	.74	11.3 (35.2)	14.1 (77.8)	0.39 (0.2 to 0.76)	.006^d^
Noncardiac clinical parameters	17 (54.8)	5 (38.5)	1.39 (0.72 to 2.7)	.32	19.6 (61.3)	8.6 (47.6)	1.32 (0.74 to 2.36)	.35
**Secondary outcomes**								
Corticosteroid course duration, median (IQR), d	10 (4 to 16)	5 (5 to 10)	6.16 (−0.13 to 12.46)	.06	10 (5 to 20)	5 (5 to 11)	6.04 (0.33 to 11.75)	.04^d^
Time to normal (≥55%) LVEF, median (IQR), d	3 (2 to 4)	2 (1.5 to 2.5)	2.19 (−1.34 to 5.72)	.22	3 (2 to 4)	2 (1 to 2)	2.46 (−0.86 to 5.78)	.14
Normal LVEF (≥55%) at discharge	103 (92.8)	63 (92.6)	1.03 (0.59 to 1.8)	.93	110.3 (94.8)	56.5 (92.4)	1.23 (0.67 to 2.24)	.50
Coronary abnormalities at discharge	5 (4.5)	3 (4.4)	0.98 (0.49 to 1.96)	.96	3.9 (3.4)	2.3 (3.7)	0.92 (0.42 to 1.99)	.83
Vasoactive medication requirement, median (IQR), d^e^	3 (1 to 4)	2 (2 to 4)	0.48 (−0.42 to 1.38)	.29	3 (1 to 4)	3 (2 to 4)	0.32 (−0.59 to 1.24)	.48
Worst pericardial effusion^f^	9 (13.8)	4 (16.0)	0.9 (0.48 to 1.67)	.73	6.2 (9.8)	4.6 (16.9)	0.73 (0.39 to 1.37)	.32
Duration of fever, median (IQR), d	6 (5 to 7.5)	5 (4 to 6)	0.59 (−0.04 to 1.22)	.07	6 (4 to 7)	5 (5 to 6)	0.35 (−0.24 to 0.94)	.19
Complications	12 (10.8)	4 (5.9)	1.34 (0.76 to 2.36)	.31	8.7 (7.5)	4.2 (6.9)	1.02 (0.57 to 1.82)	.95
Total length of stay, d	6 (4 to 8)	4 (3 to 6)	2.35 (1.43 to 3.28)	<.001^d^	6 (4 to 8)	5 (4 to 7)	1.53 (0.68 to 2.39)	.001^d^
ICU transfer ≤24 h of therapy initiation^g^	7 (18.4)	2 (4.4)	2.04 (0.95 to 4.35)	.07	7 (14.0)	0.93 (3.4)	1.82 (0.71 to 4.66)	.21
ICU length of stay, median (IQR), d^h^	5 (4 to 6)	3.5 (3 to 4)	0.93 (−1.21 to 3.06)	.34	5 (3 to 5)	3.5 (3 to 4)	0.99 (−1.08 to 3.05)	.29
Readmissions^i^	6 (5.4)	3 (4.4)	1.07 (0.55 to 2.1)	.84	4.5 (3.9)	1.5 (2.4)	1.17 (0.5 to 2.71)	.72

Abbreviations: ICU, intensive care unit; IVIG, intravenous immunoglobulin; LS-means, least square means; LVEF, left ventricular ejection fraction; OR, odds ratio.

^a^ Categorical variables are expressed as frequency (percentage) with OR, and continuous variables as median (IQR) with LS-means.

^b^ Reference was the corticosteroids group.

^c^ Among patients whose initial therapy failed.

^d^ Statistically significant.

^e^ Among patients requiring vasoactive medications.

^f^ Mild to moderate vs trivial, among patients with a pericardial effusion.

^g^ Among patients who were not in the ICU at the start of therapy.

^h^ Among patients who required ICU care.

^i^ Within 6 months of diagnosis.

#### Primary Outcome

Treatment failure rates did not significantly differ between the corticosteroids and IVIG plus corticosteroids groups (OR, 0.95; 95% CI, 0.67-1.33). Among patients whose initial therapy failed, treatment failure in the IVIG plus corticosteroids group was significantly more likely to be based on lack of improvement or worsening laboratory parameters (OR, 1.96; 95% CI, 1.07-3.60) and significantly less likely to be based on cardiovascular markers (OR, 0.39; 95% CI, 0.20-0.76), per clinician assessment.

#### Secondary Outcomes

Total median length of stay (LS-means, 1.53; 95% CI, 0.68-2.39) and median duration of corticosteroid course (LS-means, 6.04; 95% CI, 0.33-11.75) were significantly longer in the IVIG plus corticosteroids group compared with the corticosteroids group, while other secondary outcomes, including coronary abnormalities, showed no significant differences.

## Discussion

The present study compares short-term outcomes of patients with MIS-C grouped according to initial immunomodulatory treatment, including a subgroup with milder disease successfully treated with corticosteroid monotherapy. The optimal management of MIS-C remains unknown. The latest guidance statement issued by the American College of Rheumatology recommends IVIG plus corticosteroids for most patients hospitalized with MIS-C, advising a 2- to 3-week (or longer) corticosteroid taper.^4,11^ These recommendations are based on the management guidelines for KD, in which the role of both treatments has been extensively studied.^4,12,13,14^ Unlike for KD, the efficacy of these interventions in MIS-C is not clearly established, leading to practice variability across institutions.^2,4,15,16^ In our local guidance, disease severity and KD-like features influence therapy selection (eFigure in the Supplement).

Previous data suggest that the addition of corticosteroids to IVIG may facilitate recovery in MIS-C.^17,18,19^ Two large multicenter studies and a smaller retrospective analysis comparing the outcomes of patients receiving IVIG vs IVIG plus corticosteroids as first-line therapy showed that the latter group had lower risk of receiving adjunctive therapy, lower risk of cardiovascular dysfunction, and shorter ICU stays; the group receiving combined therapy had a more severe initial presentation, similar to our cohort, likely accounting for more aggressive initial treatment.^17,18,19^

Evidence regarding the role of corticosteroid monotherapy in MIS-C remains limited. A recent case series reported successful treatment of 23 of 31 patients (74.2%) with corticosteroid monotherapy, using variable doses based on the presence of cardiac involvement.^20^ A small retrospective study highlighted IVIG monotherapy as an independent risk factor for therapy failure, associated with slower recovery vs corticosteroids alone.^7^ In contrast, a large multicenter retrospective analysis found no substantial differences in recovery among patients receiving IVIG monotherapy, corticosteroid monotherapy, or IVIG plus corticosteroids as first-line MIS-C therapy, although a subanalysis limited to patients meeting World Health Organization criteria for MIS-C did show a reduction in the need for respiratory support by day 2 or later and death with corticosteroid monotherapy vs IVIG monotherapy.^21^

In our cohort, initial therapy with corticosteroids alone was associated with similar rates of treatment failure, shorter median corticosteroid course duration, and shorter median total length of stay compared with therapy with IVIG plus corticosteroids, after adjusting for baseline presentation and severity. Longer inpatient stay and corticosteroid course duration in the IVIG plus corticosteroids group may have been related to sequelae of severe disease, such as need for inpatient rehabilitation and protracted inflammatory state. Failure of initial therapy was more likely due to cardiac-related reasons and less likely due to worsening laboratory parameters in the corticosteroid monotherapy group. This finding should be interpreted with caution, as reasons for therapy failure were based on clinician assessment; furthermore, the remaining objective cardiovascular outcomes did not differ between groups after adjusted analysis. Notably, the nonuse of IVIG did not translate into worse coronary outcomes. Our findings support the use of corticosteroid monotherapy at least in a subset of patients with mild disease. Given the potential for broad immunosuppression, the courses of corticosteroids should be kept as short as possible.^3^ We demonstrate that 5- to 10-day courses were sufficient in patients with mild MIS-C.

Importantly, 19% of patients receiving corticosteroids as initial treatment required adjuvant immunosuppression, as did more than 20% of patients in the other 2 groups; this underscores the importance of continued research for optimal management, timing for escalation of treatment, and upfront disease stratification, which could guide aggressive treatment selection for patients with more severe disease, with probable reduction of treatment failure rates.

Adverse events are an important consideration for therapy selection; hence, we sought to assess the rates of adverse events that were potentially related to the medications. However, because of our study design, we were unable to control these event rates for confounding effects of frequent phlebotomies (especially in younger patients), variations in clinical judgment to transfuse, introduction of adjuvant medications, and most importantly, worsening MIS-C (potentially leading to inflammation-associated microangiopathy).^22^ Nevertheless, it is worth noting that hyperglycemia was observed in 18.6% of patients, but insulin requirement was infrequent (1.8%). The diagnosis of hemolytic anemia was rare (0.9%) in our cohort. We also examined severe anemia because some patients may have lacked complete laboratory evaluation for hemolysis, and we also found this to be uncommon (4.6%), although 11.6% of patients in this cohort received packed red blood cell transfusions. Other adverse events (except for fever during IVIG infusion) were infrequent as well. Future research designed to evaluate these events in MIS-C is needed.

Disadvantages of including IVIG therapy as part of primary treatment include high cost (especially in low-income countries), potential shortages, interference with other serological diagnoses, and need to delay live viral vaccinations with a resultant risk of reemergence of vaccine-preventable diseases. Thus, considering that corticosteroids are inexpensive and potentially effective in short courses, their use as monotherapy for mild MIS-C should be considered.^20^ Differentiating MIS-C from KD remains difficult, and patients who meet criteria for KD, particularly those at high risk of developing coronary artery aneurysms (eg, infants), should still receive IVIG as part of initial therapy.

### Limitations

Our study has limitations, mainly related to its retrospective design with the potential for selection bias. Because of its heterogeneous presentation and the lack of a standard definition for treatment failure in MIS-C, we relied on clinician judgment and documentation of the reasons for additional treatment as our primary outcome, reflecting actual clinical practice at our centers, while also evaluating objective cardiac and noncardiac clinical parameters as secondary outcomes. We addressed variability by the use of multivariate analysis and IPTW methodology, which allowed us to balance potentially confounding characteristics between our 2 largest groups and control for this potential source of bias, thus providing clear exposure-outcome associations.

Another limitation is our moderately sized cohort in a single hospital system; our IVIG monotherapy group was particularly small, which precluded us from pursuing adjusted analysis that included these patients due to being unable to balance several covariates across 3 study groups. Although multicenter studies provide the advantage of larger samples, the heterogeneity in the management protocols and MIS-C case definitions make the comparisons less straightforward. Our setting allowed us to use a uniform process for identifying cases and overall consistent institutional management protocols, considering that rapidity of recognition of MIS-C varied widely due to increased awareness over time and protocol updates generated some treatment variability.^15^ Thus, despite excluding a minority of patients receiving IVIG monotherapy from adjusted analysis, we were able to compare 2 moderately sized patient groups, obtaining results that can potentially affect current clinical practices.

## Conclusions

Initial corticosteroid monotherapy was associated with similar rates of treatment failure, shorter corticosteroid course duration, and shorter median inpatient stay compared with initial therapy with IVIG plus corticosteroids, after accounting for baseline features and disease severity. Initial therapy failure was less likely due to abnormal laboratory parameters and more likely due to abnormal cardiac parameters in patients receiving corticosteroid monotherapy vs IVIG plus corticosteroids; interpretation of these findings is challenging because they relied on the treating clinician’s interpretation and documentation. Reassuringly, objective cardiac outcomes were similar between groups.

We demonstrate that a subset of patients with mild MIS-C were successfully treated with 10 days or less of corticosteroid monotherapy, leaving need for universal IVIG use to consideration.

## Notes

Supplement. **eTable 1.** Definitions **eTable 2.** Covariates included in propensity score calculation **eTable 3.** Demographics, baseline clinical features, and initial laboratory values for all groups with unadjusted comparisons **eTable 4. **Adjuvant therapies and adverse events potentially related to medications for all groups with unadjusted comparisons **eTable 5. **Primary and secondary outcomes for all groups with unadjusted comparisons **eTable 6. **Additional echocardiographic findings at any point during illness **eTable 7. **Patients requiring re-admission after initial hospitalization: brief description and timeline **eFigure. **Current Children’s Healthcare of Atlanta algorithm for initial immunomodulatory therapy in multisystem inflammatory syndrome in children (MIS-C)
eReference
