# Supplementary material for: Short-term Outcomes of Corticosteroid Monotherapy in Multisystem Inflammatory Syndrome in Children
Source: JAMA Pediatr. 2022 Mar 28;176(6):576–84. doi: 10.1001/jamapediatrics.2022.0292 (PMC8961405; doi:10.1001/jamapediatrics.2022.0292)
Supplement: Supplement. — eTable 1. Definitions eTable 2. Covariates included in propensity score calculation eTable 3. Demographics, baseline clinical features, and initial laboratory values for all groups with unadjusted comparisons eTable 4. Adjuvant therapies and adverse events potentially related to medications for all groups with unadjusted comparisons eTable 5. Primary and secondary outcomes for all groups with unadjusted comparisons eTable 6. Additional echocardiographic findings at any point during illness eTable 7. Patients requiring re-admission after initial hospitalization: brief description and timeline eFigure. Current Children’s Healthcare of Atlanta algorithm for initial immunomodulatory therapy in multisystem inflammatory syndrome in children (MIS-C) eReference [file jamapediatr-e220292-s001.pdf]

Supplemental Online Content

Villacis-Nunez DS, Jones K, Jabbar A, et al. Short-term outcomes of corticosteroid monotherapy in multisystem inflammatory syndrome in children. *JAMA Pediatr*. Published online March 28, 2022. doi:10.1001/jamapediatrics.2022.0292

eTable 1. Definitions

eTable 2. Covariates included in propensity score calculation

eTable 3. Demographics, baseline clinical features, and initial laboratory values for all groups with unadjusted comparisons

eTable 4. Adjuvant therapies and adverse events potentially related to medications for all groups with unadjusted comparisons

eTable 5. Primary and secondary outcomes for all groups with unadjusted comparisons

eTable 6. Additional echocardiographic findings at any point during illness

eTable 7. Patients requiring re-admission after initial hospitalization: brief description and timeline

eFigure. Current Children's Healthcare of Atlanta algorithm for initial immunomodulatory therapy in multisystem inflammatory syndrome in children (MIS-C)

eReference

This supplemental material has been provided by the authors to give readers additional information about their work.

© 2022 Villacis-Nunez DS et al. *JAMA Pediatrics*.

eTable 1: Definitions

Category	Definition
**Comorbidities**	Immunosuppressive disorder, malignancy, obesity, diabetes mellitus, seizure disorder, cardiac disease, sickle cell disease, congenital malformations, lung disease, other
**System involvement**	
Gastrointestinal	Abdominal pain, vomiting, diarrhea, elevated liver enzymes, and/or elevated bilirubin
Respiratory	Shortness of breath, cough, chest pain, pneumonia, respiratory support
Neurologic	Headache, altered mental state, syncope/near syncope, meningitis, and/or encephalopathy
Renal	Acute kidney injury, and/or renal failure
Mucocutaneous	Rash, and/or mucocutaneous lesions
Ocular	Conjunctival injection, and/or periorbital edema
Hematologic	Elevated D-dimers, thrombophilia, and/or thrombocytopenia
Cardiovascular	Shock, hypotension, elevated brain natriuretic peptide or NT-pro brain natriuretic peptide, elevated troponin, left ventricular ejection fraction <55%, pericardial effusion, coronary abnormalities (z-score >2.0 in any of the coronary arteries), myocarditis, pericarditis, arrhythmia, and/or need for vasoactive medications
Other	Neck pain, drooling, trismus, myalgia, and/or cervical lymphadenopathy
**Complications**	Dialysis, thrombosis, congestive heart failure, acute respiratory distress syndrome, liver failure, and/or stroke
**Factors pertaining reasons for failure of initial therapy**	
Fever	Fever (temperature $\geq$ 38° Celsius) persistence or recrudescence documented as the reason for therapy escalation by the treating clinical in the medical record
Worsening or lack of improvement of laboratory parameters	Values out of the normal range per local laboratory reference for cell counts, ferritin, fibrinogen, C-reactive protein, D-dimer, albumin, alanine aminotransferase, and/or aspartate aminotransferase determined to be worsening or not improving by the treating clinician and documented as the reason for therapy escalation in the medical record
Worsening or lack of improvement of cardiac parameters	Left ventricular ejection fraction <55%, coronary abnormalities (z-score >2.0 in any of the coronary arteries), brain natriuretic peptide above the upper limit of normal, troponin above the upper limit of normal, vasoactive requirement, and/or hypotension determined to be worsening or not improving by the treating clinician and documented as the reason for therapy escalation in the medical record
Worsening or lack of improvement of non-cardiac clinical parameters	Mucocutaneous, ocular, neurologic, renal, respiratory and/or other (neck pain, drooling, trismus, myalgia, cervical lymphadenopathy) clinical manifestations of MIS-C determined to be worsening or not improving by the treating clinician and documented as the reason for therapy escalation in the medical record
**Factors pertaining adverse events potentially related to medications**	
Adverse events potentially related to medications	Commonly known side effects from either corticosteroid or IVIG administration, pre-defined by consensus among the authors. These were considered to be present if identified de-novo during inpatient stay
Aseptic meningitis	Headache, neck stiffness and/or meningeal signs leading to a diagnosis of aseptic meningitis documented in the medical record
Fever during IVIG infusion	Temperature $\geq$ 38° Celsius if pre-IVIG temperature was normal, or increase in temperature by 1° Celsius if pre-IVIG temperature was $\geq$38° Celsius
Hyperglycemia	Plasma glucose $\geq$200 mg/dl at least once during hospitalization.
Hypertension	Sustained blood pressure above the 99th percentile for age and height, requiring initiation of antihypertensives
Hemolytic anemia	A 2 g/dl drop in hemoglobin accompanied by a positive hemolysis marker including positive direct Coombs test or decreased haptoglobin levels, or documented new diagnosis of hemolytic anemia in medical record. Proxy markers were recorded including: hemoglobin variation, hemoglobin drop post-IVIG, packed red blood cell transfusions, and hemoglobin <7 g/dL while inpatient

© 2022 Villacis-Nunez DS et al. *JAMA Pediatrics*.

eTable 1: Definitions - continued

Category	Definition
Psychosis	New onset hallucinations or delirium
Hemoglobin variation	Initial hemoglobin minus lowest inpatient hemoglobin
Hemoglobin drop post-IVIG	Pre-IVIG hemoglobin minus lowest hemoglobin within 72 hours following IVIG administration
Nadir hemoglobin	Lowest documented inpatient hemoglobin value
**Medications**	
IVIG dosing	Standard dose: 2 g/kg
Corticosteroids	Methylprednisolone, prednisone/prednisolone, dexamethasone
Corticosteroid dosing	Doses of methylprednisolone and prednisone/prednisolone were considered equivalent. Dexamethasone was excluded from corticosteroid dose calculations. Methylprednisolone dose of 10-30 mg/kg (or 1000 mg if weight >35 kg) every 24 hours was considered pulse dosing.
**Severe inflammation**	Meeting 2 or more of the following on admission laboratories: white blood cell count >12x10^3/mL, albumin level <3 g/dL, C-reactive protein level > 10 mg/dL, ferritin level > 500 mg/dL, D-dimer units >660 ng/mL, and fibrinogen <150 mg/dL

Abbreviations: IVIG: Intravenous immunoglobulin

© 2022 Villacis-Nunez DS et al. *JAMA Pediatrics*.

eTable 2: Covariates included in propensity score calculation

Covariate	Categories
Age	Younger than 5 years vs 5 years or older
Race	African American vs Caucasian vs Other/refused
Ethnicity	Hispanic or Latino vs Non-Hispanic or Latino
Comorbidities	Present vs Absent
Time from fever onset to therapy initiation	<5 days vs $\geq$5 days
System Involvement	> 3 organ systems vs $\leq$3 organ systems
Initial platelet count	$<150x10^3$/uL vs $\geq 150x10^3$/uL
First left ventricular ejection fraction <55%	Present vs absent
Coronary abnormalities on admission	Present vs absent
Vasoactive use	Present vs absent
Intensive care status at the start of initial therapy	Present vs absent
Severe inflammation	Present vs absent

© 2022 Villacis-Nunez DS et al. *JAMA Pediatrics*.

eTable 3: Demographics, baseline clinical features and initial laboratory values for all groups with unadjusted comparisons

Baseline features	Total (n= 215)	CS Group (n= 69)	IVIG Group (n= 31)	IVIG+CS Group (n= 115)	Two-group comparisons		
					CS vs IVIG Groups	CS vs IVIG+CS Groups	IVIG vs IVIG+CS Groups
					P value	P value	P value
Age (years)	8 (5-12)	10 (6-14)	7 (5-9.5)	8 (5-12)	0.005	0.10	0.10
Male sex	135 (62.8)	42 (60.9)	22 (71.0)	71 (61.7)	0.46	1	0.46
Race/Ethnicity							
African American	118 (54.9)	30 (43.5)	23 (74.2)	65 (56.5)	0.02	0.23	0.19
Caucasian	75 (34.9)	30 (43.5)	7 (22.6)	38 (33.0)			
Other/refused[a]	22 (10.2)	9 (13.0)	1 (3.2)	12 (10.4)			
Hispanic/ Latino	47 (22.2)	18 (26.1)	4 (13.3)	25 (22.1)	0.20	0.67	0.44
Comorbidities	89 (41.4)	33 (47.8)	6 (19.4)	50 (43.5)	0.01	0.67	0.02
Obesity	64 (29.8)	24 (34.8)	4 (12.9)	36 (31.3)	0.03	0.63	0.04
Heart disease	3 (1.4)	0 (0.0)	0 (0.0)	3 (2.6)	1	0.29	0.29
Sickle cell disease	1 (0.5)	0 (0.0)	0 (0.0)	1 (0.8)	1	1	1
Lung disease	27 (12.6)	13 (18.8)	1 (3.2)	13 (11.3)	0.06	0.19	0.30
Congenital malformations	2 (0.9)	1 (1.4)	1 (3.2)	0 (0.0)	0.53	0.38	0.21
ICU status	120 (55.8)	23 (33.3)	22 (71.0)	75 (65.2)	0.001	<0.001	0.70
**System involvement**							
Gastrointestinal	206 (95.8)	66 (95.7)	29 (93.5)	111 (96.5)	0.64	1	0.61
Respiratory	136 (63.3)	34 (49.3)	22 (71.0)	80 (69.6)	0.07	0.01	1
Neurologic	125 (58.1)	37 (53.6)	13 (41.9)	75 (65.2)	0.39	0.16	0.03
Renal	53 (24.7)	7 (10.1)	10 (32.3)	36 (31.3)	0.02	0.002	1
Mucocutaneous	124 (57.7)	34 (49.3)	17 (54.8)	73 (63.5)	0.76	0.08	0.50
Ocular	150 (69.8)	40 (58.0)	23 (74.2)	87 (75.7)	0.18	0.02	1
Hematologic	212 (98.6)	67 (97.1)	31 (100.0)	114 (99.1)	1	0.65	1
Cardiovascular	204 (94.9)	61 (88.4)	29 (93.5)	114 (99.1)	0.72	0.004	0.11
Initial LVEF (%)	60.05 (52.92-66.73)	63.34 (57.79-69.23)	59.04 (53.03-64.44)	58 (50-64.8)	0.02	0.001	0.67
Initial LVEF <55%	61 (28.4)	12 (17.4)	9 (29.0)	40 (34.8)	0.29	0.02	0.70
Coronary abnormalities	11 (5.2)	3 (4.4)	1 (3.2)	7 (6.2)	1	0.86	1
Pericardial effusion	85 (39.5)	18 (26.1)	16 (51.6)	51 (44.3)	0.02	0.02	0.60
Vasoactive drug use	86 (40.0)	11 (15.9)	12 (38.7)	63 (54.8)	0.02	<0.001	0.17
Arrhythmia	14 (7.7)	5 (10.0)	1 (4.0)	8 (7.4)	0.66	0.81	1
Other	102 (47.4)	35 (50.7)	15 (48.4)	52 (45.2)	1	0.57	0.91
Fever onset to therapy initiation (days)	4 (3-6)	4 (3-6)	5 (4.5-6)	4 (3-6)	0.01	0.58	0.02
Symptom onset to therapy initiation (days)	5 (4-6)	5 (4-6)	6 (5-7.5)	5 (4-6)	0.028	0.968	0.012

© 2022 Villacis-Nunez DS et al. *JAMA Pediatrics*.

eTable 3: Demographics, baseline clinical features and initial laboratory values for all groups with unadjusted comparisons - continued

Baseline features	Total (n= 215)	CS Group (n= 69)	IVIG Group (n= 31)	IVIG+CS Group (n= 115)	Two-group comparisons		
					CS vs IVIG Groups	CS vs IVIG+CS Groups	IVIG vs IVIG+CS Groups
					P value	P value	P value
**Initial laboratory values[b]**							
Hemoglobin (g/dl)	12 (11.1-12.7)	12.4 (11.2- 12.9)	11.7 (10.9-12.35)	11.9 (11.2-12.70)	0.02	0.30	0.13
WBC (10^3/uL)	9.42 (7.1-12.43)	9.36 (7.01-12.03)	9.79 (7.64-13.97)	9.36 (6.87-12.37)	0.40	0.96	0.30
RNC (% of WBC)	78 (69.75-85.35)	76 (68.5-84.4)	76 (70.5-84.55)	80.9 (70.5-85.7)	0.94	0.16	0.35
RLC (% of WBC)	10 (6.5-16)	10 (5.72-18.70)	11 (7-13.5)	10.05 (6.12-14.23)	0.98	0.37	0.48
Platelets (10^3/uL)	166 (130-217)	193 (145-244)	180 (119.5-229)	149 (125.5-202.5)	0.17	0.001	0.34
Albumin (g/dL)	2.9 (2.5-3.2)	3 (2.7- 3.4)	2.7 (2.3-2.95)	2.9 (2.45-3.2)	0.002	0.06	0.07
Sodium (mmol/L)	134 (131-136)	134 (132-137)	133 (131-135.5)	134 (131- 136)	0.17	0.17	0.63
AST (U/L)	42 (26-75)	36 (24-72)	50 (26-66)	43 (28-77)	0.20	0.09	0.82
ALT (U/L)	37 (25-56.5)	33 (24-48)	39 (28.5-57.5)	39 (25-59)	0.2	0.14	0.86
CRP (mg/dL)	13.9 (9.9-18.6)	13.6 (10.2-16.8)	15.1 (8.15-17.35)	13.9 (9.75-20.7)	0.90	0.43	0.57
ESR (mm/hour)	41 (31-62)	42 (29.75-58.5)	55 (38.5-66.5)	40 (30.75-59.25)	0.09	0.06	0.97
D-dimer units (ng/mL)	1599.5 (908.75-2423.5)	1092.5 (610.25-1760.75)	2118 (1595-2804.5)	1631 (1126.5- 2534.5)	<0.001	0.001	0.11
Ferritin (mg/dL)	475.23 (249.95-918.43)	317.87 (212.18-592.05)	624.57 (286.76-1142.14)	513.87 [270- 1103.4)	0.02	0.003	0.68
Fibrinogen (mg/dL)	554 (456-651)	568 (500.5-654)	526 (448- 619)	523 (446- 652.75)	0.18	0.12	0.75
BNP (pg/mL)	165.2 (47.15-559.95)	77.8 (14.7-377.3)	389.35 (113.97-1031)	232.5 (62.7- 567.6)	0.008	0.004	0.29
Troponin (ng/mL)	0.19 (0.04-0.72)	0.23 (0.08-0.58)	0.1 (0.04-1.03)	0.18 (0.04-0.72)	0.52	0.59	0.70
+ ANA	12 (22.2)	2 (20.0)	3 (27.3)	7 (21.2)	1	1	0.69
+ SARS-CoV-2 IgG	207 (98.6)	64 (97.0)	31 (100.0)	112 (99.1)	1	0.56	1
+ SARS-CoV-2 PCR	73 (34.4)	29 (43.3)	5 (16.1)	39 (34.2)	0.02	0.29	0.08

Categorical variables are expressed as frequency (percentage), and continuous variables as median (interquartile range).
[a]Other race categories include: American Indian, Alaska Native or Aboriginal Canadian, Native Hawaiian, Other Pacific Islander, Asian
[b]First documented value in each laboratory category
Abbreviations: ALT: Alanine aminotransferase; ANA: Antinuclear antibodies; AST: Aspartate aminotransferase; BNP: Brain natriuretic peptide; CRP: C-Reactive protein; ESR: Sedimentation rate; ICU: Intensive care unit; IgG: Immunoglobulin G; LVEF: Left ventricular ejection fraction; PCR: Polymerase chain reaction; RLC: Relative lymphocyte count; ANC: RNC: Relative Neutrophil count SARS-CoV-2: Severe acute respiratory syndrome coronavirus 2; U: Units; WBC: White blood cell count; +: Positive

© 2022 Villacis-Nunez DS et al. *JAMA Pediatrics*.

eTable 4: Adjuvant therapies and adverse events potentially related to medications for all groups with unadjusted comparisons

Category	Total (n= 215)	CS Group (n= 69)	IVIG Group (n= 31)	IVIG+CS Group (n= 115)	Two-group comparisons		
					CS vs IVIG Groups	CS vs IVIG+CS Groups	IVIG vs IVIG+CS Groups
					P value	P value	P value
Initial corticosteroid dose (mg/kg/day)	1.9 (1.3-2)	1.9 (1-2)	1.9 (1.8-1.95)	1.95 (1.4-2)	0.48	0.07	0.68
Maximum corticosteroid dose (mg/kg/day)	2 (1.55-2.1)	1.9 (1.1-2)	1.9 (1.85-2)	2 (1.67-2.2)	0.52	0.002	0.24
**Adjuvant therapies**							
**Immunomodulators (among patients failing initial therapy)**							
First IVIG	10 (15.9)	10 (76.9)	0 (0.0)	0 (0.0)	NA	NA	NA
Second IVIG	23 (36.5)	1 (7.7)	3 (17.6)	19 (57.6)	NA	NA	0.008
Anakinra	7 (11.1)	0 (0.0)	1 (5.9)	6 (18.2)	1	0.24	0.45
Tocilizumab	2 (3.2)	0 (0.0)	0 (0.0)	2 (6.1)	1	0.92	0.54
Infliximab	2 (3.2)	1 (7.7)	0 (0.0)	1 (3.0)	0.43	1	1
Corticosteroids (not pulse dosing)	13 (20.6)	0 (0.0)	13 (76.5)	0 (0.0)	NA	NA	NA
Pulse dose corticosteroids	17 (27.0)	4 (30.8)	2 (11.8)	11 (33.3)	0.36	1	0.19
Miscellaneous[a]	19 (30.2)	3 (23.1)	0 (0.0)	16 (48.5)	0.07	0.21	0.002
**Antiplatelet and anticoagulant agents**							
Low dose aspirin	197 (91.6)	64 (92.8)	27 (87.1)	106 (92.2)	0.45	1	0.48
High dose aspirin	43 (20.0)	1 (1.4)	14 (45.2)	28 (24.3)	<0.001	<0.001	0.03
Enoxaparin	169 (78.6)	51 (73.9)	18 (58.1)	100 (87.0)	0.16	0.04	0.001
Heparin	8 (3.7)	1 (1.4)	0 (0.0)	7 (6.1)	1	0.26	0.35
**Adverse events potentially related to medications**							
Aseptic meningitis	3 (1.4)	0 (0.0)	0 (0.0)	3 (2.6)	1	0.29	1
Fever during IVIG infusion	53 (34.0)	1 (10.0)[b]	16 (51.6)	36 (31.3)	0.03	0.29	0.06
First infusion[c]	50 (32.1)	1 (10.0)	16 (51.6)	33 (28.7)	0.03	0.28	0.03
Second infusion[d]	4 (17.4)	0 (0.0)	0 (0.0)	4 (21.05)	NA	NA	1
Hyperglycemia	40 (18.6)	7 (10.1)	5 (16.1)	28 (24.3)	0.51	0.03	0.47
Insulin requirement	4 (1.9)	2 (2.9)	0 (0.0)	2 (1.7)	1	0.63	1
Hypertension	10 (4.7)	2 (2.9)	2 (6.5)	6 (5.2)	0.77	0.71	1
Psychosis	6 (2.8)	2 (2.9)	0 (0.0)	4 (3.5)	1	1	0.58
Gastrointestinal bleeding	9 (4.2)	2 (2.9)	1 (3.2)	6 (5.2)	1	0.71	1
Hemolytic anemia	2 (0.9)	0 (0.0)	0 (0.0)	2 (1.7)	1	0.71	1
Hemoglobin variation (g/dL)	2.3 (1.3-3.25)	1.5 (1- 2.3)	2 (1.35-3.55)	2.7 (1.9- 3.6)	0.008	<0.001	0.37
Hemoglobin drop post-1st IVIG (g/dL)[c]	1.6 (0.8-2.33)	0.9 (0.4-1.2)	1.5 (1-2.23)	1.8 (0.8-2.5)	0.03	0.002	0.99
Hemoglobin drop post-2nd IVIG (g/dL)[d]	0.5 (0-1.6)	NA	1 (0.6-1.4)	0.5 (-0.1-1.5)	NA	NA	0.84

© 2022 Villacis-Nunez DS et al. *JAMA Pediatrics*.

eTable 4: Adjuvant therapies and adverse events potentially related to medications for all groups with unadjusted comparisons - continued

Category	Total (n= 215)	CS Group (n= 69)	IVIG Group (n= 31)	IVIG+CS Group (n= 115)	Two-group comparisons		
					CS vs IVIG Groups	CS vs IVIG+CS Groups	IVIG vs IVIG+CS Groups
					P value	P value	P value
Hemoglobin ≤7 g/dl at any point	10 (4.6)	1 (1.4)	3 (9.7)	6 (5.2)	0.09	0.26	0.40
PRBC transfusion	25 (11.6)	2 (2.9)	9 (29.0)	14 (12.2)	<0.001	0.03	0.04
Nadir hemoglobin (g/dL)	9.5 (8.6-10.6)	10.6 (9.7-11.4)	8.7 (8.05-9.9)	9.3 (8.4-9.9)	<0.001	<0.001	0.20

Categorical variables are expressed as frequency (percentage), and continuous variables as median (interquartile range).

[a]Miscellaneous adjuvant therapies included hydroxychloroquine (n=1), increase corticosteroid dose (not pulse dosing) (n=12); re-initiation of corticosteroid therapy for symptom recurrence after discontinuation (n=4); switch to methylprednisolone from dexamethasone (n=2).

[b]Among IVIG recipients (n=10) [c]Among recipients of first IVIG dose [d]Among recipients of second IVIG dose

Abbreviations: IVIG: Intravenous Immunoglobulin; NA: Non-applicable/unable to calculate; PRBC: Packed Red Blood Cells.

© 2022 Villacis-Nunez DS et al. *JAMA Pediatrics*.

eTable 5: Primary and secondary outcomes for all groups with unadjusted comparisons

Outcome	Total (n= 215)	CS Group (n= 69)	IVIG Group (n= 31)	IVIG+CS Group (n= 115)	Two-group comparisons		
					CS vs IVIG Groups	CS vs IVIG+CS Groups	IVIG vs IVIG+CS Groups
					P value	P value	P value
**Primary outcome:** Failure of initial therapy	63 (29.3)	13 (18.8)	17 (54.8)	33 (28.7)	0.001	0.19	0.01
Reasons for failure of initial therapy[a]							
Fever	46 (73.0)	11 (84.6)	12 (70.6)	23 (69.7)	0.43	0.46	1
Laboratory parameters	39 (61.9)	7 (53.8)	11 (64.7)	21 (63.6)	0.82	0.78	1
Cardiac parameters	32 (50.8)	7 (53.8)	10 (58.8)	15 (45.5)	1	0.85	0.55
Non-cardiac clinical parameters	29 (46.0)	5 (38.5)	6 (35.3)	18 (54.5)	1	0.51	0.32
**Secondary outcomes**							
Duration of corticosteroids (days)	9 (5-15)	5 (5-10)	8 (5-16)	10 (5-17.5)	0.33	0.001	0.53
Cardiovascular outcomes							
Time to normal (≥55%) LVEF (days)	3 (2-4)	2 (1.75- 2.25)	4 (2.75-5)	3 (2-4)	0.005	0.03	0.08
LVEF <55% at discharge	14 (6.5)	5 (7.2)	1 (3.2)	8 (7.0)	0.66	1	0.68
Coronary abnormalities at discharge	8 (3.7)	3 (4.3)	0 (0.0)	5 (4.3)	0.55	1	0.55
Vasoactive drug duration (days)	0 (0-2)	0 (0-0)	0 (0-2.5)	1 (0-3)	0.01	<0.001	0.34
Worst pericardial effusion[b]							
Trivial	92 (84.4)	21 (84.0)	15 (78.9)	56 (86.2)	0.71	0.75	0.48
Mild to moderate	17 (15.6)	4 (16.0)	4 (21.1)	9 (13.8)			
Duration of fever (days)	6 (5-7)	5 (4-6)	6 (6-7)	6 (5-7.5)	<0.001	0.02	0.09
Complications	16 (7.4)	4 (5.8)	0 (0.0)	12 (10.4)	0.31	0.42	0.07
Pulmonary embolism	1 (0.5)	1 (1.4)	0 (0.0)	0 (0.0)	1	0.38	1
Congestive heart failure	3 (1.4)	1 (1.4)	0 (0.0)	2 (1.7)	1	1	1
ARDS	13 (6.0)	3 (4.3)	0 (0.0)	10 (8.7)	0.55	0.38	0.12
Total length of stay (days)	5 (4-7)	4 (3- 6)	6 (4.5-8)	6 (4-8)	<0.001	<0.001	0.84
ICU transfer within 24 hours of therapy start[c]	11 (11.6)	2 (4.3)	0 (0.0)	9 (22.5)	1	0.02	0.18
ICU length of stay (days)	3 (0-5)	0 (0-3)	5 (0-5.5)	3 (2-5)	<0.001	<0.001	0.76
Re-admissions[d]	10 (4.6)	3 (4.3)	0 (0.0)	7 (6.1)	0.55	0.74	0.35

Categorical variables are expressed as frequency (percentage), and continuous variables as median (interquartile range).
[a]Among patients failing initial therapy [b]Among patients with a pericardial effusion [c]Among patients who were not in the intensive care unit at the start of therapy
[d]Within 6 months after hospitalization
Abbreviations: ARDS: Acute respiratory distress syndrome: ICU: Intensive care unit; LS-means: Least square means; LVEF: Left ventricular ejection fraction

© 2022 Villacis-Nunez DS et al. *JAMA Pediatrics*.

eTable 6: Additional echocardiographic findings at any point during illness

Category	Total (n= 215)	CS Group (n= 69)	IVIG Group (n= 31)	IVIG+CS Group (n= 115)	Two-group comparisons		
					CS vs IVIG Groups	CS vs IVIG+CS Groups	IVIG vs IVIG+CS Groups
					P value	P value	P value
Worst recorded LVEF (%)	54.88 (46.13-61.04)	59.18 (53- 64.98)	55 (45.29-60)	52 (41.45-57.75)	0.011	<0.001	0.238
LVEF <55% at any point	109 (50.7)	21 (30.4)	15 (48.4)	73 (63.5)	0.132	<0.001	0.188
Coronary abnormalities at any point	26 (12.1)	5 (7.2)	5 (16.1)	16 (13.9)	0.277	0.232	0.775

Categorical variables are expressed as frequency (percentage), and continuous variables as median (interquartile range).
Abbreviations: LVEF: Left ventricular ejection fraction

© 2022 Villacis-Nunez DS et al. *JAMA Pediatrics*.

eTable 7: Patients requiring re-admission after initial hospitalization: Brief description and timeline

Patient	Initial therapy Group	Reason for re-admission and additional description
1	IVIG+CS	Encephalopathy and psychosis due to SARS-CoV-2 induced neuroinflammatory syndrome 11 days post-completion of 1-week CS taper; required additional 6-week CS taper
2	IVIG+CS	MIS-C symptom recurrence 24 hours after completion of 5-day CS course; required CS for 2 additional weeks
3	IVIG+CS	Acute gastroenteritis 3 days post-completion of 5-day CS course; MIS-C therapy not restarted, improved with supportive care
4	IVIG+CS	Non-cardiac chest pain due to gastritis vs costochondritis 5 days post-discharge; completed scheduled last CS dose inpatient (11-day course); no additional MIS-C therapy needed
5	IVIG+CS	MIS-C symptom recurrence and coronary artery aneurysm 19 days post-discharge (11 days post-completion of 19-day CS taper); required pulse corticosteroids followed by prolonged CS taper + infliximab[1]
6	IVIG+CS	Bacterial pneumonia presenting 8 weeks post-discharge (6 weeks post-completion of 23-day CS taper), treated with antibiotics
7	IVIG+CS	MIS-C symptom recurrence 24 hours after finishing 5-day CS course; required CS for 4 additional weeks
8	CS	Dehydration due to herpes simplex virus stomatitis 24 hours post-discharge while still on CS for MIS-C; MIS-C therapy continued (total CS course: 6 days)
9	CS	Urosepsis and new diagnosis of idiopathic intracranial hypertension (chronic headaches, exacerbated during infection) 4 weeks post-discharge (3 weeks post-completion of 6-day CS course). No additional MIS-C therapy needed
10	CS	MIS-C symptom recurrence 2 days after 1-week CS course; required CS for 12 additional days

Abbreviations: CS: Corticosteroids; IVIG: Intravenous Immunoglobulin; MIS-C: Multisystem inflammatory syndrome in children

© 2022 Villacis-Nunez DS et al. *JAMA Pediatrics*.

eFigure: Current Children's Healthcare of Atlanta algorithm for initial immunomodulatory therapy in Multisystem Inflammatory Syndrome in Children (MIS-C)

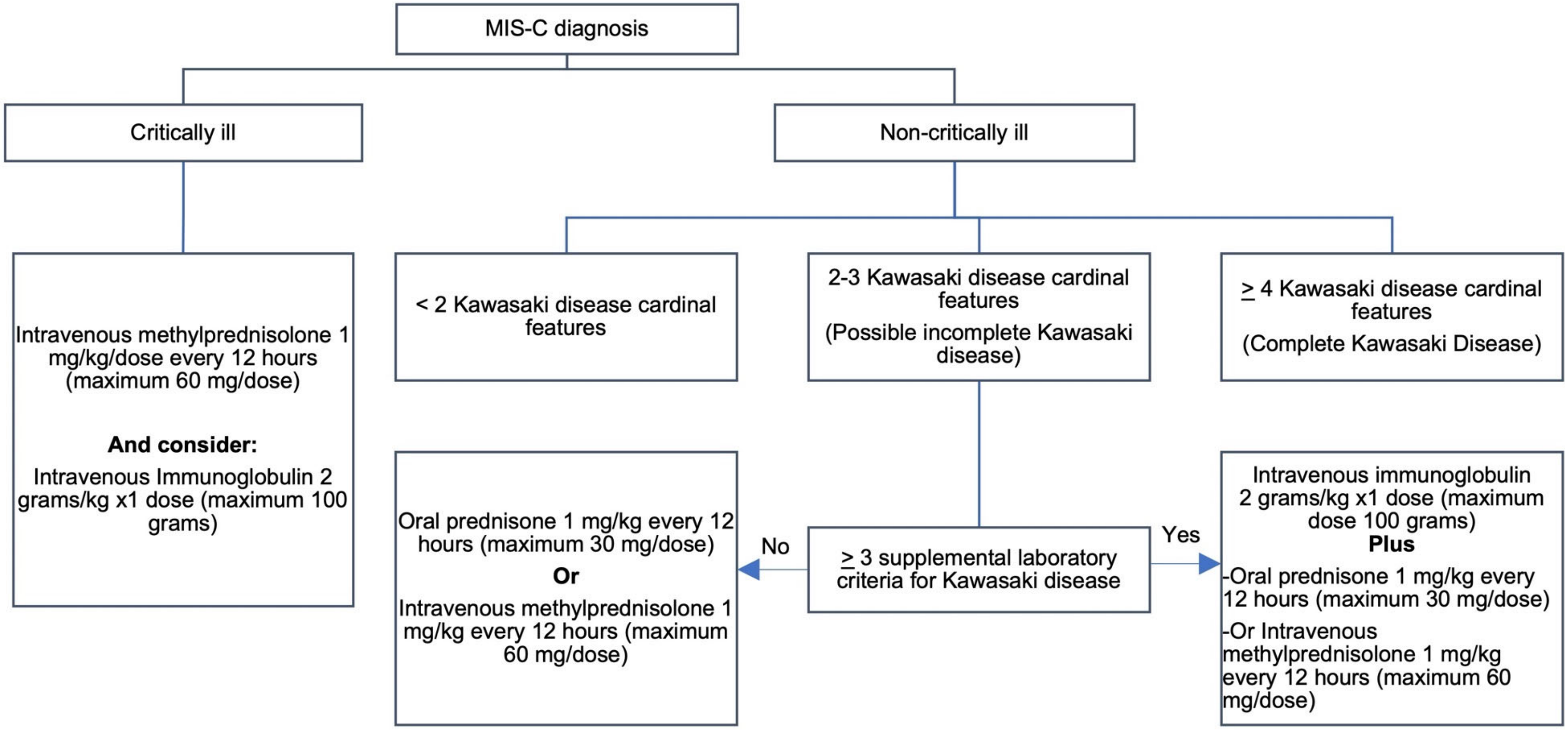

© 2022 Villacis-Nunez DS et al. *JAMA Pediatrics*.

eReference

1. Villacis-Nunez DS, Hashemi S, Nelson MC, et al. Giant Coronary Aneurysms in Multisystem Inflammatory Syndrome in Children Associated With SARS-CoV-2 Infection. JACC Case Rep. October 2021 2021;3(13)doi:10.1016/j.jaccas.2021.06.043

© 2022 Villacis-Nunez DS et al. *JAMA Pediatrics*.
